# Additive Manufacture of Three Dimensional Nanocomposite Based Objects through Multiphoton Fabrication

**DOI:** 10.3390/polym8090325

**Published:** 2016-09-01

**Authors:** Yaan Liu, Qin Hu, Fan Zhang, Christopher Tuck, Derek Irvine, Richard Hague, Yinfeng He, Marco Simonelli, Graham A. Rance, Emily F. Smith, Ricky D. Wildman

**Affiliations:** 1 Faculty of Engineering, The University of Nottingham, University Park, Nottingham NG7 2RD, UK; eaxyl3@exmail.nottingham.ac.uk (Y.L.); qin.hu@nottingham.ac.uk (Q.H.); fan.zhang@nottingham.ac.uk (F.Z.); christopher.tuck@nottingham.ac.uk (C.T.); derek.irvine@nottingham.ac.uk (D.I.); richard.hague@nottingham.ac.uk (R.H.); yinfeng.he@nottingham.ac.uk (Y.H.); marco.simonelli@nottingham.ac.uk (M.S.); 2 Nanoscale and Microscale Research Centre, The University of Nottingham, University Park, Nottingham NG7 2RD, UK; graham.rance@nottingham.ac.uk (G.A.R.); emily.smith@nottingham.ac.uk (E.F.S.)

**Keywords:** multiphoton fabrication (MF), two-photon lithography (TPL), nanocomposite, gold nanoparticles, polymerisation, metal reduction, 3D printing, additive manufacturing

## Abstract

Three-dimensional structures prepared from a gold-polymer composite formulation have been fabricated using multiphoton lithography. In this process, gold nanoparticles were simultaneously formed through photoreduction whilst polymerisation of two possible monomers was promoted. The monomers, trimethylopropane triacrylate (TMPTA) and pentaerythritol triacrylate (PETA) were mixed with a gold salt, but it was found that the addition of a ruthenium(II) complex enhanced both the geometrical uniformity and integrity of the polymerised/reduced material, enabling the first production of 3D gold-polymer structures by single step multiphoton lithography.

## 1. Introduction

Additive Manufacturing (AM) is attractive due to its design freedoms, particularly with relation to increased levels of complexity [1]. An extension of AM is additive fabrication at the nanoscale, putting into reach length scales that are useful for manipulating, for example, electromagnetic radiation at optical wavelengths; this nano-scale AM is most commonly achieved using femtosecond laser induced multiphoton fabrication (MF) [2,3,4,5]. MF was first demonstrated for the fabrication of three-dimensional (3D) structures by Maruo in 1997 [6], resulting in features at subwavelength scales [7,8]. Since this first demonstration, MF has been used to create complexity for a range of applications, including photonic crystals, mechanical devices and microscopic models [9,10,11]. MF can be utilised with a wider range of photochemistries than simply for polymerisation. For example, Kaneko et al. [2] reported gold nanoparticle grating lines fabricated using metal ion-doped polyvinyl alcohol (PVA), and in 2006, a self-standing silver gate structure with electrical conductivity was obtained using a metal-ion aqueous solution by Tanaka et al. [12]. This process was further enhanced by the addition of a suitable dye that improved metallic reduction and enabled the use of reduced laser powers [13,14,15,16,17,18]. These advances offer the potential to produce periodic metallic nano/microstructures all in a single processing step [19].

Recent endeavours have been focused on extending the AM approach to multiple materials [20,21,22,23,24], and this has been complemented by efforts to process nanocomposites using MF. Combined metal reduction and polymerisation has been observed using a range of methods, but each requires multiple processing stages [4,5,17,19,25,26]. A significant step forward, however, is in the single-step fabrication by MF of nano-composites, reducing the need for multiple washes and fabrication steps. This has been demonstrated, in two dimensions, by simultaneous photopolymerisation and photoreduction [25], and, in a similar fashion, by the creation of three dimensional filigree-like conductive elements that are ‘glued’ together with a polymer resin [27].

In this paper, however, a method for fabricating truly three-dimensional nanocomposite objects will be presented. Following the lead of Shukla et al. [13], it will be shown that, by suitable selection of monomer, initiator and dye, a stable three-dimensional object can be formed. In this case, a radical polymerisation is utilised, whilst taking advantage of previously observed beneficial effects of adding ruthenium complexes on multiphoton reactivity [21], leading to the role of the initiator in overcoming the competition between polymerisation and reduction being explored.

## 2. Materials and Methods

### 2.1. General Approach

Formulations able to realise three-dimensional multiphoton fabricated structures were identified in the following way. First, acrylate based formulations were prepared with combinations of monomer, photoinitiator, gold(III) chloride hydrate, and photosensitive dye. These formulations were systematically varied; and then trials with a commercial MF system were performed to identify the fidelity of two- and three-dimensional structures. Structures successfully formed were then characterised to confirm the presence of gold within the polymeric framework.

### 2.2. Multiphoton Fabrication

A commercial two-photon lithography facility (Nanoscribe GmbH Photonic Professional GT) was utilised for the MF. The system is driven by a fibre laser at a 780 nm central wavelength, 80 MHz repetition rate and a 120-fs pulse duration. The laser beam was focused using an oil immersion objective lens (1.4 NA, 63×, 190 µm WD). Structures were built by moving the sample position in the *XY* plane using a galvo mirror and in the *Z* direction using a piezoelectric actuator to move the objective. The laser power was varied between 10 and 50 mW and the scan speed was 10,000 µm/s.

**Figure 1:**
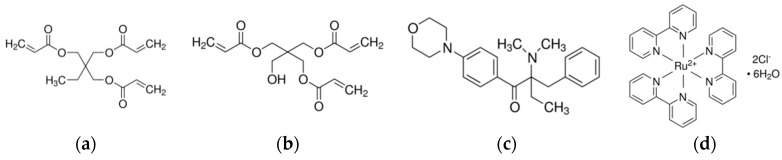
Chemical structures of (**a**) trimethylopropane triacrylate (TMPTA); (**b**) pentaerythritol triacrylate (PETA); (**c**) 2-benzyl-2-(dimethylamino)-4′-morpholinobutyrophenone (DBMP); and (**d**) ruthenium(II) complex.

Two monomers, trimethylopropane triacrylate (TMPTA) (as received, Sigma-Aldrich, Dorset, UK) and pentaerythritol triacrylate (PETA) (as received, Sigma-Aldrich, Dorset, UK) were used as base materials for the proposed formulations. 2-benzyl-2-(dimethylamino)-4′-morpholinobutyrophenone (DBMP) (as received, Sigma-Aldrich, Dorset, UK) was used as the photoinitiator and tris(2,2′-bipyridyl) dichlororuthenium(II) hexahydrate (bipy) (as received, Sigma-Aldrich, Dorset, UK) was used as a dye. The chemical structures are shown in Figure 1. Gold(III) chloride hydrate, HAuCl_4_·*3*H_2_O (Sigma-Aldrich, Dorset, UK) was used as the metal salt and ethanol was employed as a solvent for both the ruthenium(II) and gold(III) chloride hydrate.

### 2.3. Material Preparation

To prepare materials ready for fabrication the following steps were taken. Firstly, the gold chloride hydrate and ruthenium(II) complex were separately dissolved in ethanol at a maximum concentration of 0.2 g/mL. Subsequently, combinations of 3 wt % DBMP, 3 wt % of gold(III) chloride hydrate and 0.1 wt % of Ru(II) complex were added to the two monomers, resulting in 6 formulations (Table 1). Ethanol was assumed to be fully evaporated during the writing process and is not shown in Table 1. The additives were mixed using a magnetic stirrer operating at a speed of 600 rpm for at least 30 min. Oxygen is known to affect the quality of final structures (and polymerisation in general) so, as a consequence, a separate set of experiments were performed to assess the effect of purging by passing nitrogen through the mixtures for 15 min. A drop of each of the resultant combinations was deposited on a glass slide with a pipette, which was then loaded onto the Nanoscribe for TPL processing. After processing, the sample on the glass slide was developed by washing in propylene glycol monomethyl ether acetate (PGMEA) (Sigma-Aldrich, Dorset, UK), 2-propanol (Sigma-Aldrich) and water to remove unreduced gold salt and residual monomer. As a final step, the samples were dried in air. Elemental analysis was performed using energy-dispersive X-ray spectroscopy (EDX) (Philips XL30, Philips, Guildford, UK) and X-ray photoelectron spectroscopy (XPS) (Kratos Axis Ultra DLD, Kratos Analytical Ltd, Manchester, UK), chemical analysis by Raman spectroscopy (Horiba–Jobin–Yvon LabRAM, Horiba–Jobin–Yvon, Palaiseau, France) and scanning electron microscopy (SEM) was used to perform morphology inspection (Philips XL30, Philips, Guildford, UK).

## 3. Results

### 3.1. Fabrication of 2D and 3D Polymeric Objects

**Figure 2:**
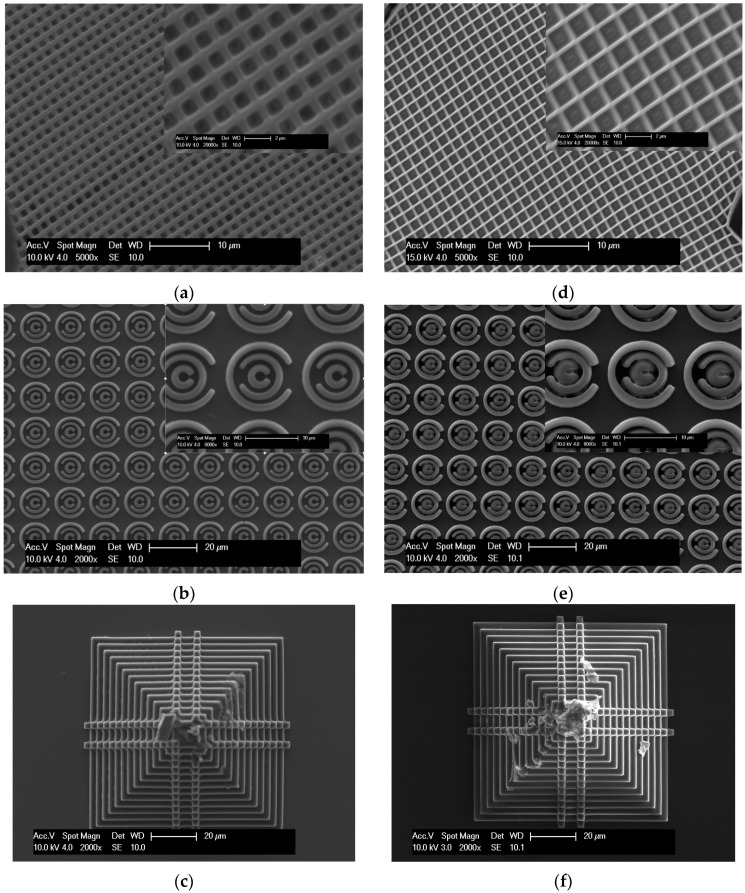
SEM images of 2D and 3D fabricated structures using Formulations 1 and 4. (**a**–**c**) show the results of fabricating increasingly more complex structures with Formulation 1 (Table 1); (**d**–**f**) show the results of fabricating increasingly more complex structures with Formulation 4.

A series of experiments were performed to ascertain whether simple two- and three-dimensional structures could be manufactured using the proposed formulations. Figure 2 shows the results of a series of fabrications from the monomer only that is able to produce designs of increasing complexity. Although 0.2 wt % DBMP in TMPTA and PETA is sufficient for polymerisation, 3 wt % was used to provide consistency across all formulations. The minimum laser output power for each is given in Table 2, and was varied to optimise the feature size and avoid bubble generation due to heat formation. Figure 2a,d illustrates that sub-micron feature sizes are possible with both TMPTA and PETA, and that more complex structures are possible in two-dimensions (2D) (Figure 2b,e) and in 3D (Figure 2c,f).

### 3.2. The Effect of Adding Gold Salt on the Integrity of Structures

Having established the suitability of the base formulation for MF, gold(III) chloride hydrate (3 wt %) was added, and an assessment of the amount of photoinitiator required was performed. By increasing the concentration of the photoinitiator in steps of 1 wt % and attempting to fabricate structures, it was determined that 3 wt % was the minimum required concentration of photoinitiator for TMPTA with 3 wt % gold chloride hydrate. It is postulated that the limited number of free radicals led to competition between free radical polymerisation and metal reduction with the generated free radicals preferentially reducing the gold ions, thus suppressing polymerisation. Polymerisation was only observed when the concentration of DBMP was at least equal to the concentration of gold chloride hydrate (3 wt %). When the gold chloride hydrate concentration was raised to 5 wt %, it was necessary to add an equivalent wt % of photoinitiator in order to obtain polymer formation. It is likely, however, that this correspondence is a coincidence and further work is required to identify the exact mechanism of competition between the two photochemistries.

**Figure 3:**
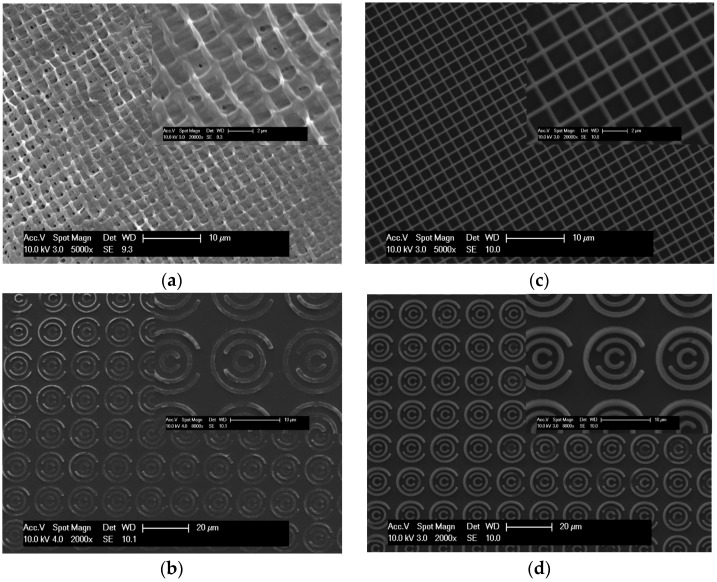

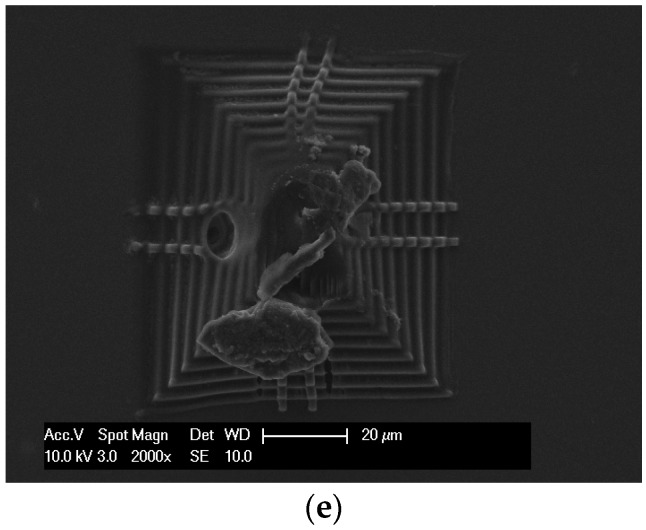
SEM images of 2D and 3D fabricated structures using Formulations 2 and 5. (**a**,**b**) show the results of fabricating increasingly more complex structures with Formulation 2 (Table 1); (**c**–**e**) show the results of fabricating increasingly more complex structures with Formulation 5.

When considering the fidelity of the fabrication, it was noted that even though TMPTA achieved polymerisation, the line quality was consistently poor (see Figure 3a), resulting in build failures as the complexity of the object to be fabricated was increased (Figure 3b). When using PETA during equivalent experiments, a markedly improved overall performance was observed, with consistent 2D structures fabricated (Figure 3c,d). The grid lines of Formulation 5 (Figure 3c) were more uniform compared to Formulation 2 (Figure 3a) and the line width compared well with a minimum observable size of about 300 nm when using monomer only. However, despite these promising results, the fabrication of 3D structures was a challenge, as deformations appeared as the build was increased in the *Z* direction (Figure 3e). Inspecting the moieties present in the monomers, it is noted that difference between PETA and TMPTA lies in the presence of the hydroxyl group in PETA, which is likely to result in a different distribution of electron density compared to TMPTA, which will have a direct impact on the reactivity and consequently on *T_g_*, all of which will impact on pattern quality.

### 3.3. The Effect of Adding a Two Photon Sensitive Dye on the Integrity of Structures

**Figure 4:**
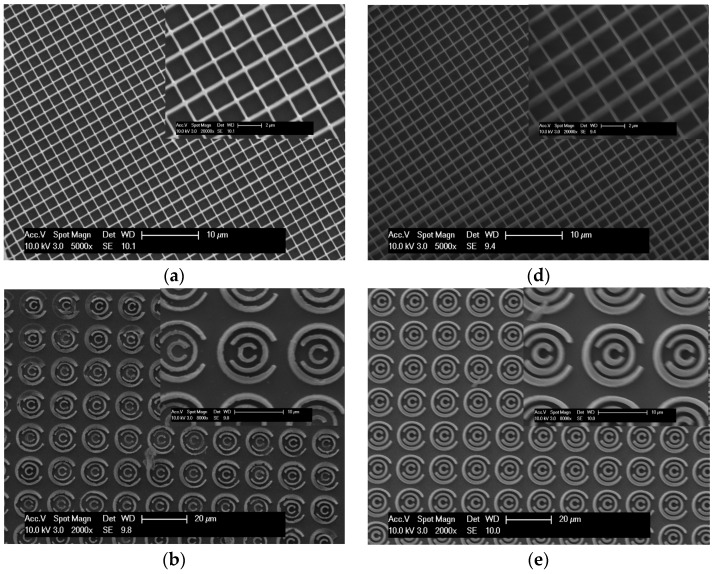

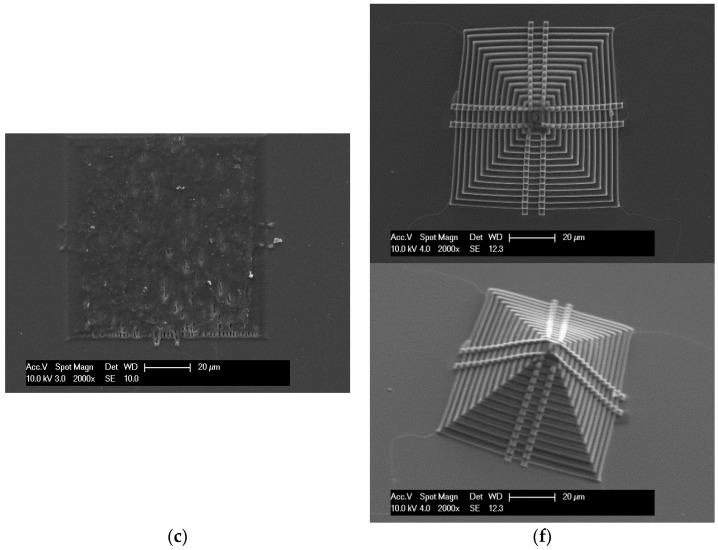
SEM images of 2D and 3D fabricated structures using Formulations 3 and 6. (**a**–**c**) show the results of fabricating increasingly more complex structures with Formulation 3 (Table 1); (**d**–**f**) show the results of fabricating increasingly more complex structures with Formulation 6; (**f**) shows both plan view and at a tilt angle of 30°.

Ruthenium(II) complexes have been reported previously as effective additions for promoting gold reduction [16] and thus 0.1 wt % tris(2,2′-bipyridyl) dichlororuthenium(II) hexahydrate was added to the gold(III) chloride hydrate containing formulations (Table 1). Figure 4 shows SEM images of structures fabricated based on Formulations 3 and 6 (Table 1).

The addition of the dye to Formulation 3 results in a significantly improved grid structure (Figure 4a). The role of ion [Ru (bipy)_3_]^2+^ has previously been cited as producing a large two-photon cross section [28], whereby it strongly absorbs the IR light and results in a triplet metal-to-ligand charge-transfer (^3^MLCT) excited state, which transfers and locates electrons on bipy ligands to help gold reduction and polymerisation [16,29]. Although fabrication of a 3D structure was possible with PETA, the addition of the Ru(II) complex was not sufficient to allow 3D fabrication with the TMPTA based formulation (Figure 4c).

**Figure 5:**
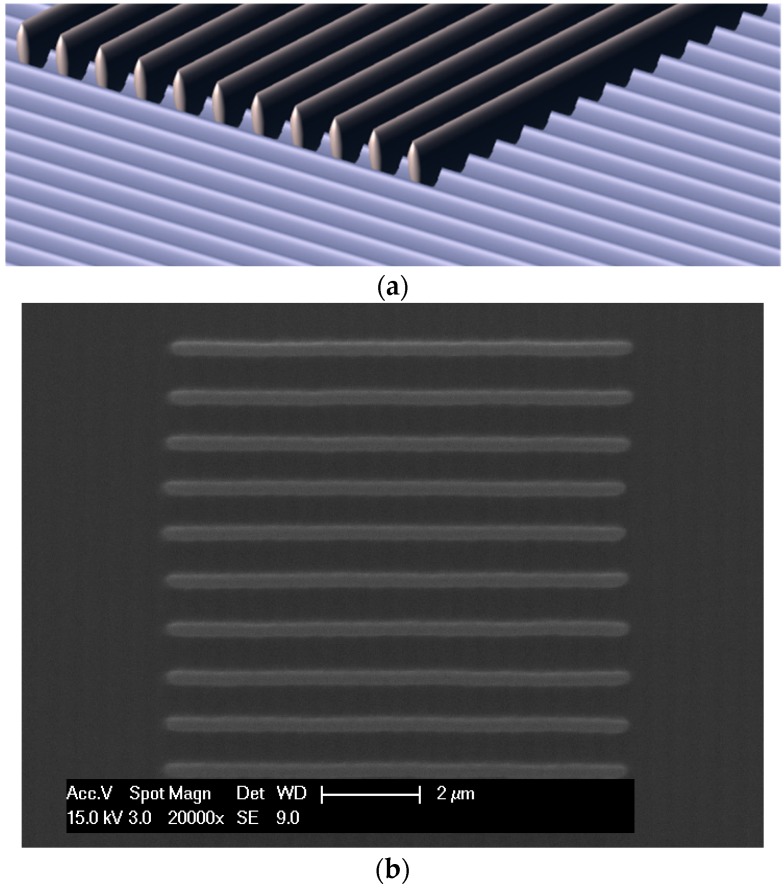
(**a**) A protocol for ensuring level lines was developed by creating an initial set of parallel features, on to which lines for inspection were fabricated; and (**b**) a typical set of fabricated lines (Formulation 6 without degassing, 35 mW).

**Figure 6:**
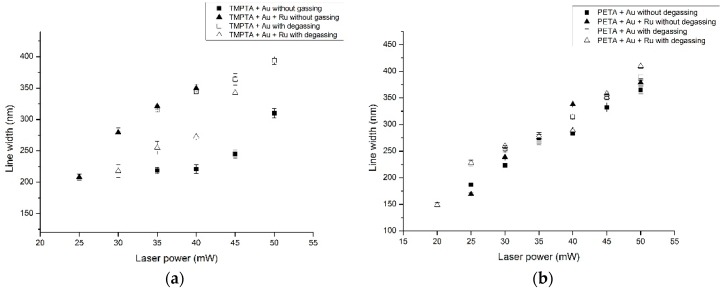
(**a**) Line width as a function of laser power for Formulation 2 and 3, with and without degassing and (**b**) line width as a function of laser power for Formulation 5 and 6, with and without degassing.

To examine further the processing differences for each gold containing formulation (2, 3, 5 and 6), single lines were written using laser powers ranging from 10 to 50 mW in increments of 0.5 mW (Figure 5a,b). From SEM images of the resulting fabrications, the line width was measured and shown in Figure 6. It can be observed that without Ru(II) and without degassing results in the smallest line width (~150 nm) using PETA. Degassing only had a significant effect when the dye was not present, and that the effect was greater for TMPTA than for PETA based formulations. It is likely that the reduction in line width, though welcome, is a result of a reduction in the number of radicals available for polymerisation propagation, since the presence of oxygen will consume a proportion of free radicals. This will have the effect of increasing the threshold intensity required for two-photon absorption, narrowing the region of the laser beam able to drive polymerisation. It can be seen therefore that there are positive consequences of an absence of the dye and removal of oxygen, but it must be counterbalanced with the observation that adding the Ru(II) complex tends to promote more stable structures and enables fabrication of 3D objects. A further experiment was performed to identify the minimum laser powers required to initiate fabrication (Table 2). This further emphasises the role that the addition of the dye has on eliminating detrimental effects associated with the presence of oxygen, whilst also illustrating the relative ease with which PETA based materials can be fabricated in comparison to TMPTA.

Raman spectra of samples 2, 3, 5 and 6 and of the monomers were obtained and the degree of conversion (DC) for the samples was calculated using the ratios of areas of the peaks associated with displacement of the C=C and C=O bonds in the polymer materials (not shown) [30]. Two distinctive peaks were observed at 1638 and 1726 cm^−1^, which represent the C=C and C=O groups respectively. The C=C bonds were reduced in order as part of a Michael addition transformation and resolved to C–C bonds during polymerisation resulting in a reduction of intensity at 1638 cm^−1^, whilst the intensity of C=O is unaffected due to the non-participation of this bond in the reactions. The degree of conversion, Δ, can thus be calculated from:

(1) $$\Delta =1-\frac{A_{C=C}/A_{C=O}}{A_{C=C}^{\prime }/A_{C=O}^{\prime }}$$

where $A_{c=c}$, $A_{C=O}$, $A_{c=c}^{\prime }$ and $A_{C=O}^{\prime }$ are the peak intensities in the Raman spectra related to the C=C and C=O groups in the polymerised structures and the non-polymerised monomers. This relies on the assumption that disproportionated C=C bonds are not produced, but it is likely that they are present and as a consequence the calculation of the degree of conversion represents an estimate of the lower bound. Using Equation (1), the estimated degrees of conversion for each of the samples produced from the formulations were calculated and are shown in Table 3. This indicated a greater degree of conversion for PETA, correlating with observations of greater structural integrity for this monomer.

### 3.4. Characterisation of the Presence of Gold within Gold-Polymer Nanocomposites

**Figure 7:**
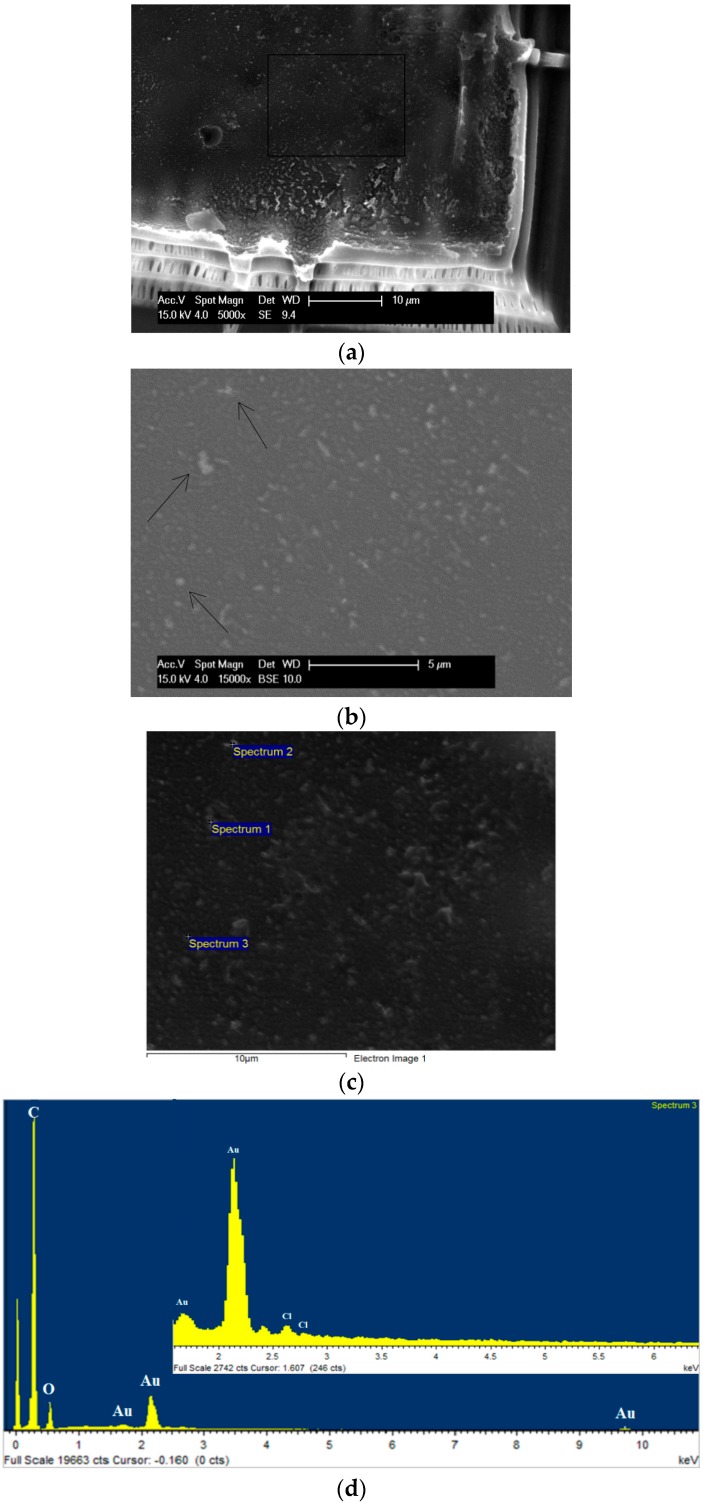
(**a**) Scanning electron microscope (SEM) image of a part completed 3D fabrication using Formulation 5; (**b**) a backscattered electron (BSE) image of the fabricated structure from (**a**) showing the location of suspected gold particles (arrows indicate location of particles) at higher magnification; (**c**) SEM image of Energy dispersive X-ray spectroscopy (EDX) of the area; and (**d**) EDX spectrum of the particles at location 3.

**Figure 8:**
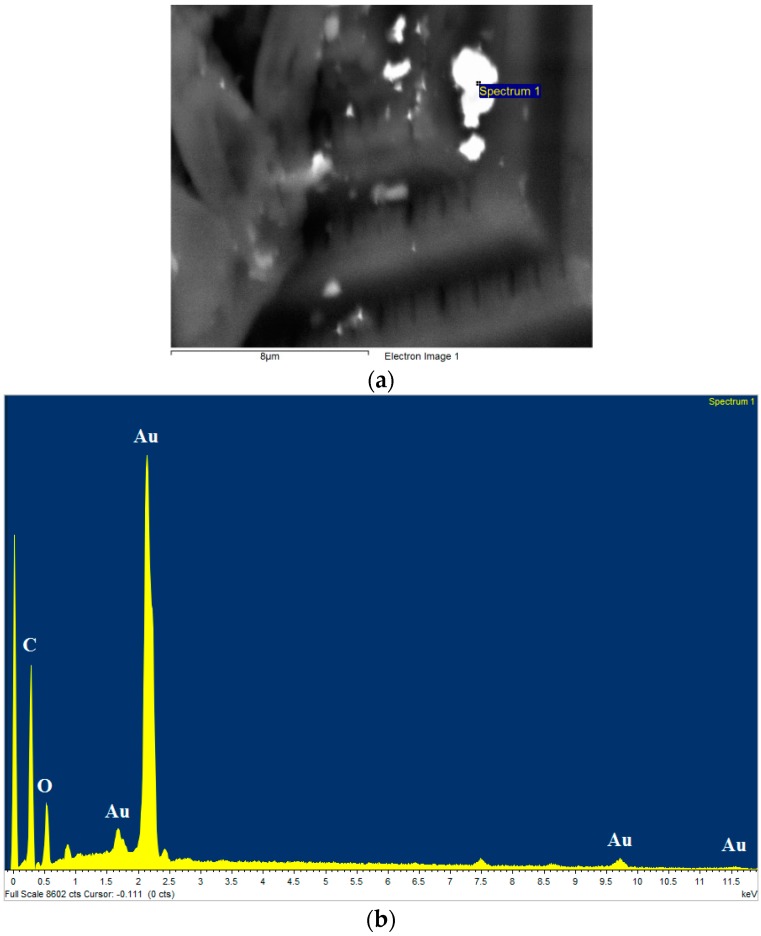
(**a**) A backscattered electron (BSE) image showing the presence of suspected gold particles at the surface of a structure fabricated using Formulation 6; (**b**) an energy dispersive X-ray spectroscopy (EDX) spectrum of the particle located at Position 1 in (**a**), indicating the presence of elemental gold.

**Figure 9:**
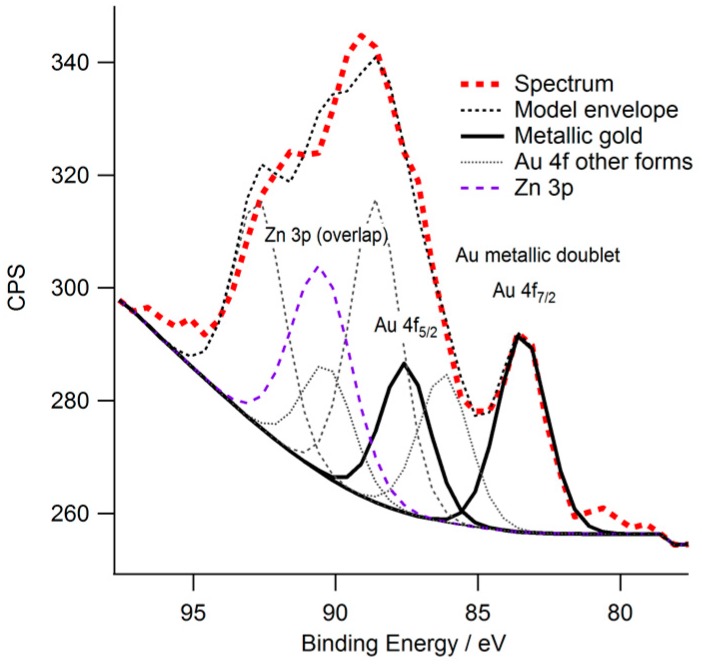
An X-ray photoelectron spectroscopy spectrum determined for a partially completed three dimensional build of Formulation 5. The peak at 83.6 +/− 0.2 eV was identified as signaling the presence gold nanoparticles proximal to the exposed surface. The remainder of the peaks are associated with gold in other, unreduced, states and with Zn from the surrounding substrate glass.

To confirm the presence of gold within the 3D structure (Formulation 5, Figure 3e), a build was halted midway during the fabrication of the pyramid structure. Secondary electron (SE), backscattered electron (BSE) SEM and images from EDX of the semi-fabricated structures were then obtained (Figure 7). Gold particles were observed from the BSE images (Figure 7b) and gold was detected using EDX from the flat surface (Figure 7c). The spectra of particles at locations 1, 2 and 3 (Figure 7c) showed two clear Au peaks at 2.2 and 9.7 eV (shown for location 3 only in Figure 7d). A Cl peak was determined at around 2.8 eV. A qualitative comparison was then performed between the expected area ratio (~1:4, Gold to Chlorine) and the observed area ratio of the main peaks. The observed area ratio of the main peaks for each element was found to be 5.3:1 (Gold to Chlorine), strongly pointing towards the formation of elemental gold. A similar inspection of a 3D object fabricated using formulation 6 (Figure 8) shows the presence of elemental gold through a large Au peak at 2.2 eV with no significant Cl peak. Further, XPS analysis showed the presence of a very small amount of gold (<0.1 atomic %) in the sample surface (Figure 9). XPS is a surface sensitive technique which excites photoelectrons that have relatively short attenuation lengths of 3–5 nm in polymers, therefore the information depth for XPS analysis is about 10 nm for a typical 1000 eV photoelectron excited by the standard Al K alpha X-ray source (1486.6 eV). A large area of analysis approximately 300 × 700 microns was used so some signal was observed from the surrounding substrate. High sensitivity/low resolution spectra at pass energy 80 eV were used to obtain the Au 4f energy region in an attempt to ascertain if gold was present in the surface. The Au 4f peak for bulk gold is normally observed at 84 eV [31,32]; a broad feature was observed at higher binding energy, which may be associated with the unreacted starting material gold chlorides, and a smaller feature was observed at 83.6 +/− 0.2 eV which is likely to be the Au 4f 7/2 peak usually associated with the Au metallic state for the nanoparticles. From these techniques, therefore it can be concluded that there is a small amount of metallic gold in the surface region, and that these are likely to be nanoparticle sized Au.

## 4. Conclusions

A comprehensive comparison of MF based additive manufacturing of nanocomposites based around two triacrylate based monomers of similar structure has been performed. It has been shown that the addition of a two photon sensitive dye, in this case tris(2,2′-bipyridyl) dichlororuthenium(II) hexahydrate can stabilise the fabrication process, allowing for the production of three-dimensional structures formed from gold-polymer nanocomposites. The presence of gold was confirmed through SEM, XPS and EDX. The two monomers were of a similar structure, with a difference in the ‘fourth arm’ being the presence of a methyl and a hydroxyl group for TMPTA and PETA respectively. Fabrication feature sizes and minimum power requirements indicated more efficient and stable processing when using PETA, which was realised in the production of truly three-dimensional nanocomposite structures.

## Figures and Tables

**Table 1:** Composition of 6 formulations, based on trimethylopropane triacrylate (TMPTA) and pentaerythritol triacrylate (PETA) monomer systems.

	Monomer	Gold Chloride Hydrate wt %	DBMP wt %	Ru(II) wt %
1	TMPTA	0	3	0
2	TMPTA	3	3	0
3	TMPTA	3	3	0.1
4	PETA	0	3	0
5	PETA	3	3	0
6	PETA	3	3	0.1

**Table 2:** The minimum laser output power (mW) for each formulation.

	Without Degassing	With Degassing
Formulation 2	25.5	22.5
Formulation 3	22.0	22.0
Formulation 5	24.0	21.5
Formulation 6	20.0	20.0

**Table 3:** Calculated DC values Formulation 2, 3, 5 and 6.

Formulation	Degree of Conversion, Δ%
2	41.6
3	44.5
5	49.6
6	49.1

## Abbreviations

The following abbreviations are used in this manuscript:

2D	2-dimensional
3D	3-dimensional
AM	Additive Manufacturing
BSE	Backscattered electron
DBMP	2-benzyl-2-(dimethylamino)-4′-morpholinobutyrophenone
EDX	Energy-dispersive X-ray
MF	Multiphoton fabrication
PETA	Pentaerythritol triacrylate
PGMEA	Propylene glycol monomethyl ether acetate
PVA	Polyvinyl alcohol
SEM	Scanning electron microscopy
TMPTA	Trimethylopropane triacrylate
TPL	Two-photon lithography
XPS	X-ray photon spectroscopy
